# Comparative Transcriptome Analysis of Gene Expression and Regulatory Characteristics Associated with Different Bolting Periods in *Spinacia oleracea*

**DOI:** 10.3390/genes15010036

**Published:** 2023-12-26

**Authors:** Hao Wu, Zhilong Zhang, Zhiyuan Liu, Qing Meng, Zhaosheng Xu, Helong Zhang, Wei Qian, Hongbing She

**Affiliations:** 1 State Key Laboratory of Vegetable Biobreeding, Institute of Vegetables and Flowers, Chinese Academy of Agricultural Sciences, Beijing 100081, China; 2 College of Life Sciences, Henan Normal University, Xinxiang 453007, China

**Keywords:** spinach, bolting, RNA-seq, differentially expressed genes (DEGs)

## Abstract

Bolting is a symbol of the transition from vegetative to reproductive growth in plants. Late bolting can effectively prolong the commercial value of spinach and is of great importance for spinach breeding. Bolting has complex regulatory networks, and current research on spinach bolting is relatively weak, with specific regulatory pathways and genes unclear. To clarify the regulatory characteristics and key genes related to bolting in spinach, we conducted a comparative transcriptome analysis. In this study, 18 samples from three periods of bolting-tolerant spinach material 12S3 and bolting-susceptible material 12S4 were analyzed using RNA-seq on, resulting in 10,693 differentially expressed genes (DEGs). Functional enrichment and co-expression trend analysis indicated that most DEGs were enriched in the photoperiod pathway, the hormone signaling pathway, and the cutin, suberin, and wax biosynthetic pathways. According to the weighted gene co-expression network analysis (WGCNA), *SpFT* (*SOV4g003400*), *SOV4g040250*, and *SpGASA1* (*SOV6g017600*) were likely to regulate bolting through the gibberellin and photoperiod pathways, and *SpELF4* (*SOV1g028600*) and *SpPAT1* (*SOV4g058860*) caused differences in early and late bolting among different cultivars. These results provide important insights into the genetic control of bolting in spinach and will help elucidate the molecular mechanisms of bolting in leafy vegetables.

## 1. Introduction

Spinach (*Spinacia oleracea* L.), which belongs to the Amaranthaceae family in the Caryophyllales order, is a diploid (2n = 2x = 12) economically important cool-season leafy vegetable crop [1,2] that is favored by consumers due to its abundant iron content (4–6 mg per 100 g dry wt) and rich variety of nutrients, such as β-carotene (provitamin A), vitamins of the B group, ascorbic acid, folates, and vitamin C [1,3]. In contemporary times, healthy and low-calorie diet habits have led to a huge demand for organic spinach production. In 2021, the total global production quantity of spinach was 32.29 million tons (http://www.fao.org/faostat/en/#datade, accessed on 10 November 2023).

Bolting is a phenomenon of flower stem elongation that occurs before flowering due to the influence of endogenous hormones and external environmental changes, such as development, age, plant hormones, photoperiod, and temperature [4]. This is one of the indicators of the transition from the vegetative stage to the reproductive stage in plants. Spinach is a dioecious species with an XY sex-determination system [5]. Bolting leads to the development of coarse and bitter leaves that are less suitable for consumption. Consequently, the late bolting trait has remained a primary objective in spinach breeding endeavors. Research on bolting mechanisms holds significant importance in extending the shelf life and increasing the yield of spinach.

The processes of bolting and flowering involve intricate pathways orchestrated by a combination of endogenous hormones and environmental factors [6]. These signals form a complex regulatory network and molecular machinery that collectively govern the coordination and control of these developmental events. Multiple studies have revealed flowering-associated signaling pathways and regulatory networks in the model plant *Arabidopsis thaliana*. The elucidated pathways underlying the processes of bolting and flowering encompass several key modalities, including photoperiod, gibberellin (GA), temperature (vernalization), autonomous, age-related, and ambient temperature pathways. In addition, there are integration factors for bolting and flowering that interact with these six pathways [7,8,9].

In the photoperiod pathway, *FLOWERING LOCUS T* (*FT*), *FLOWERING LOCUS C* (*FLC*), *CONSTANS* (*CO*), *SUPPRESSOR OF OVER-EXPRESSION OF CONSTANS 1* (*SOC1*), and other genes commonly appear during the bolting and flowering processes of Arabidopsis [6]. *FLC* inhibits bolting and flowering by encoding the MADS-box protein in the vernalization pathway [10]. *FT* is a crucial mobile signal known as “florigen” that activates meristem identity genes and *SOC1*. *SOC1* is upregulated by *FT* and downregulated by *FLC*. *CO* is the key gene in the photoperiodic pathway and belongs to *CONSTANS-LIKE* (*COL*) proteins called B-box (BBX) proteins [11]. It acts upstream of *FT* and can simultaneously activate *FT* and *SOC1* to regulate bolting and flowering under long-day (LD) conditions [6].

The GA pathway interacts with other flowering genetic pathways and phytohormone signaling pathways through either DELLA proteins or by mediating GA homeostasis [12]. The DELLA protein family serves as a negative regulatory factor in the gibberellic acid (GA) signaling pathway, capable of suppressing gene expression within the GA pathway, thereby inhibiting plant growth [13]. MicroRNA156 (miR156) and its target gene, *SQUAMOSA PROMOTER BINDING PROTEIN LIKE* (*SPL*), play pivotal roles in the age-related pathway [14]. The overexpression of miR156 delays flowering in plants [15,16], while genes such as *SPL3* and *SPL9* are involved in the transition of floral meristem identity and the shift toward reproductive growth in plants [14].

Using transcriptome data to identify bolting and flowering genes is a common research approach. Tang et al. [17] analyzed transcriptome data during *Capsicum annuum* L. flower development, showed significant levels of enrichment of transcription factor families, such as *AP2-ERF*, *MADS-box*, *MYB*, *bHLH*, and *NAC*, and identified 17 ABCDE model candidate genes in pepper. Nie et al. [18] used RNA-seq technology to analyze differential gene expression during the transition from vegetative to reproductive growth in radish (*Raphanus sativus* L.). They speculated that the transcriptional regulation of several floral integrators, including *FT*, *CO*, *SOC 1*, *FLC*, and *LFY*, integrated signals from various pathways to regulate bolting and flowering in radish. In spinach, Abolghasemi et al. [19] conducted RNA-seq on early and late bolting varieties at both the vegetative and reproductive stages. They discovered multiple differentially expressed genes (DEGs) associated mainly with the signaling pathways of vernalization, photoperiod/circadian clock, gibberellin, autonomous, and aging. They suggested that genes such as *Fructose-1*,*6-bisphosphate aldolase*, *TREHALOSE-6-PHOSPHATE SYNTHASE 1*, *FLOWERING PROMOTING FACTOR 1*, *EARLY FLOWERING*, *GIGANTEA*, and MADS-box proteins potentially play a role in initiating or delaying bolting [19]. In comparison to the previous study on bolting in spinach, our research focuses on analyzing and comparing multiple time periods. This expanded scope allows us to provide a more detailed understanding of regulatory pathways and co-expression trends, proposing more specific gene interaction networks and identify key genes. Here, we applied RNA-seq technology to analyze the gene expression differences between a bolting-tolerant material and a bolting-susceptible material at three stages, from the nutritional stage to the reproductive stage. A total of 10,693 DEGs were isolated. Gene Ontology (GO) and Kyoto Encyclopedia of Genes and Genomes (KEGG) pathway enrichment analyses revealed that multiple DEGs were closely related to the photoperiod pathway. Key genes such as *SOV6g017600*, *SOV4g003400*, and *SOV4g058860* were identified by co-expression analysis. Related analysis indicated that *FLOWERING LOCUS T*, *Gibberellin-regulated protein 1*, *EARLY FLOWERING*, and the *GRAS* domain were involved in the spinach bolting process. These results provide an important theoretical basis for the genetic regulation of bolting in spinach and help reveal the molecular regulatory mechanisms of bolting in leafy vegetables.

## 2. Materials and Methods

### 2.1. Plant Materials

This research used two inbred lines, 12S3 and 12S4, with significantly different bolting characteristics as materials. Based on previous investigations of phenotypic data, the average bolting time for 12S3 is 62 days, classifying it as a bolting-tolerant material, while 12S4 had an average bolting time of 46.5 days, classifying it as a bolting-susceptible material. Both genotypes exhibit a stable genetic background and structure, ensuring a high degree of genetic purity. All materials are the property of the Spinach Breeding Group, Vegetable and Flower Research Institute, Chinese Academy of Agricultural Sciences (Beijing, China).

**Figure 1:**
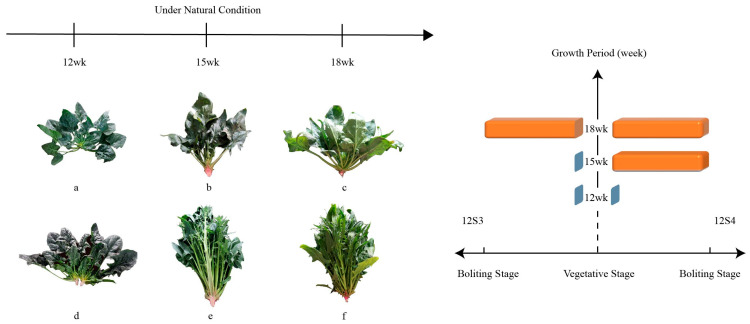
The bolting situation varied between the sampling periods of 12S3 and 12S4. (**a**) L4-12S3, unbolted; (**b**) L5-12S3, unbolted; (**c**) L6-12S3, bolted; (**d**) L4-12S4, unbolted; (**e**) L5-12S4, bolted; (**f**) L6-12S4, bolted.

These seeds were sown at the end of December 2020 in a greenhouse at the Vegetable and Flower Research Institute. We counted three weeks as one stage and we randomly collected fresh leaves of 12S3 and 12S4 at the 4th, 5th, and 6th stages, corresponding to 12, 15, and 18 weeks, respectively, for Illumina sequencing (Figure 1). At each time point, we collected three replicates for each material. Bolting-susceptible material 12S4 bolted at the 5th stage (week 15), and bolting-tolerant material 12S3 bolted at the 6th stage (week 18). All samples were immediately frozen in liquid nitrogen and stored at −80 °C.

### 2.2. RNA Extraction and Sequencing

Total RNA was extracted using the RNAprep Pure Plant Kit (Tiangen, Beijing, China) according to the manufacturer’s instructions. RNA concentration and purity were measured using NanoDrop 2000 (Thermo Fisher Scientific, Wilmington, NC, USA). RNA integrity was assessed using the RNA Nano 6000 Assay Kit of the Agilent Bioanalyzer 2100 system (Agilent Technologies, Santa Clara, CA, USA). A total amount of 1 μg RNA per sample was used as input material for the RNA sample preparations. Sequencing libraries were generated using Hieff NGS Ultima Dual-mode mRNA Library Prep Kit for Illumina (Yeasen Biotechnology Co., Ltd., Shanghai, China) following manufacturer’s recommendations. All of the operations were conducted with three repeats.

The libraries were sequenced on an Illumina NovaSeq platform to generate 150 bp paired-end reads. The Illumina raw data were submitted to the China National Center for Bioinformation (CNCB) with project ID PRJCA022070. The raw reads were further processed with the bioinformatic pipeline tool BMKCloud (www.biocloud.net, accessed on 10 November 2023) online platform.

### 2.3. RNA-Seq Analysis

Raw RNA-seq reads were filtered using fastp (v0.23.3) [20] with the parameter “−q 20”. Then, the Q20, Q30, GC content, and sequence duplication levels of the clean data were calculated. Clean reads were aligned to the spinach reference genome Monoe-Viroflay [21] using HISAT2 (v2.8.2) with default parameters [22]. Read counts were estimated using feature counts (v2.0.1) [23]. The Monoe-Viroflay sequences genome were obtained from SpinachBase website (http://www.spinachbase.org, accessed on 10 November 2023) [24]. This version had been released in December 2021.

Differential expression analysis of the two groups was performed using DESeq2 (v1.30.1) [25]. DESeq2 provides statistical routines for determining differential expression in digital gene expression data using a model based on a negative binomial distribution. The resulting *p* values were adjusted using Benjamini and Hochberg’s approach to control the false discovery rate. Genes with an adjusted *p*-value < 0.01 and fold change ≥ 2 found by DESeq2 were assigned as differentially expressed.

### 2.4. GO and KEGG Pathway Enrichment Analyses

GO enrichment analysis of differentially expressed genes was performed using the clusterProfiler package based on the Wallenius non-central hypergeometric distribution [26]; the KEGG pathway was analyzed using clusterProfiler software (v4.4.4) based on the KOBAS database [27].

### 2.5. Analysis of Co-Expression Trends and Co-Expression Networks

Co-expression trend analysis was performed on 18 samples using the K-means [28] method in the Python package. The RNA-seq data were analyzed to construct gene co-expression networks using the R package WGCNA (v4.2.3) [29].

### 2.6. qRT-PCR Analysis and Statistical Analysis

The RNA that was extracted from each sample was reverse-transcribed into cDNA using HiScript III All-in-One RT SuperMix Perfect (Vazyme, Nanjing, China), and a qRT-PCR was performed using Taq Pro Universal SYBR qPCR Master Mix (Vazyme, Nanjing, China) using an ABI Real-time System (ABI Q1 System). The primer design was completed using Primer3 software (http://frodo.wi.mit.edu/primer3/, accessed on 10 November 2023) (Supplementary Table S2). Actin was considered as the internal control, and the gene expression data were analyzed using the 2^−∆∆Ct^ method [30]. SPSS v26.0 (SPSS, Chicago, IL, USA) was used to conduct a one-way analysis of variance (ANOVA) with a Duncan’s multiple range post hoc test, and there was a significance threshold of *p* < 0.05.

## 3. Results

### 3.1. RNA Sequencing and Identification of Transcripts

We obtained a total of 118.70 Gb of clean data, with all samples reaching 6.09 Gb and Q30 base percentages of 93.29% or above. HISAT2 was used to align clean reads of each sample to the spinach reference genome Monoe-Viroflay [21], with alignment efficiency ranging from 92.17% to 97.61% (Supplementary Table S1). A total of 3421 new genes were discovered, of which 1472 were functionally annotated.

**Figure 2:**
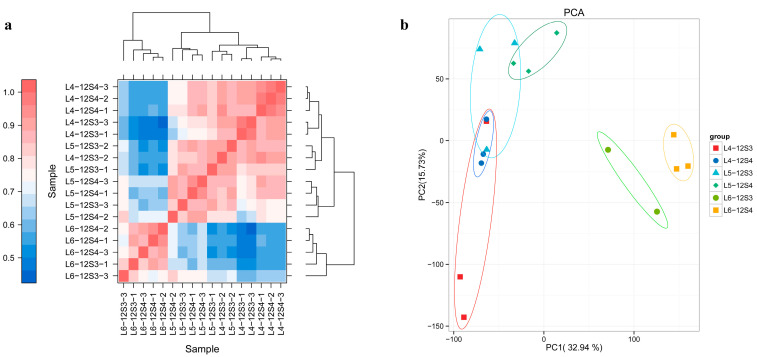
Repeated correlation assessment and principal component analysis. (**a**) Pearson correlation coefficients for comparisons among all samples; (**b**) principal component analysis based on all expressed genes, showing six distinct groups of samples.

The Pearson correlation coefficient of normalized reads per kilobase per million mapped reads (RPKM) values between samples ranged from 0.82 to 0.97 (R > 0.8) (Figure 2a), indicating high repeatability between samples within the group. These results indicate that high-quality transcriptome data were obtained for further analysis. Through principal component analysis (PCA), multiple variables were reduced to a few independent variables (i.e., principal components). The PCA diagram between the samples in this project showed good dispersion among the groups (Figure 2b). Bolted and unbolted samples were clustered at different positions according to different periods, showing significant differences.

### 3.2. Identification and Enrichment Analysis of DEGs between Bolted and Unbolted Individuals

**Figure 3:**
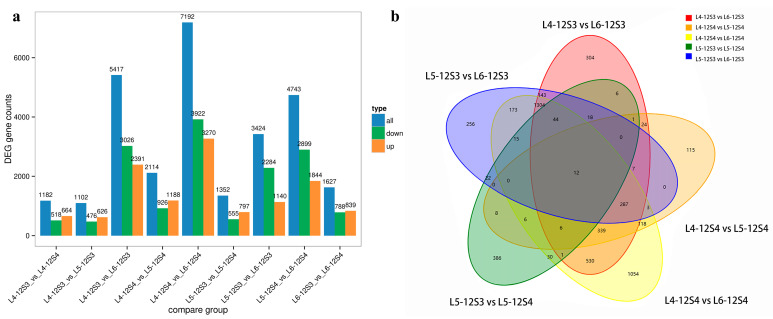
Identification of DEGs in different comparison groups. (**a**) Numbers of up- and downregulated DEGs in nine comparisons; (**b**) Venn diagram of DEGs in five comparisons between unbolted and bolted groups.

We had six groups of samples, each representing the stage of the material. Comparisons were made between every two groups to obtain all differentially expressed genes (including upregulated and downregulated), as shown in Figure 3a. A total of 10,693 DEGs were identified among nine comparison groups, most of which (9867 DEGs, 92.27%) were differentially expressed between the bolted and unbolted sample groups.

Unbolted sample groups included L4-12S3, L5-12S3, and L4-12S4, and bolted sample groups included L6-12S3, L5-12S4, and L6-12S4. The unbolted sample group was compared with the bolted sample group in pairs to obtain five comparison groups (Figure 3b). There were 7192 (3270 up- and 3922 downregulated), 5417 (2391 up- and 3026 downregulated), 3424 (1140 up- and 2284 downregulated), 2114 (1188 up- and 926 downregulated), and 1352 (797 up- and 555 downregulated) DEGs in the L4-12S4 vs. L6-12S4, L4-12S3 vs. L6-12S3, L5-12S3 vs. L6-12S3, L4-12S4 vs. L5-12S4, and L5-12S3 vs. L5-12S4 comparisons, respectively (Figure 3a,b).

**Figure 4:**
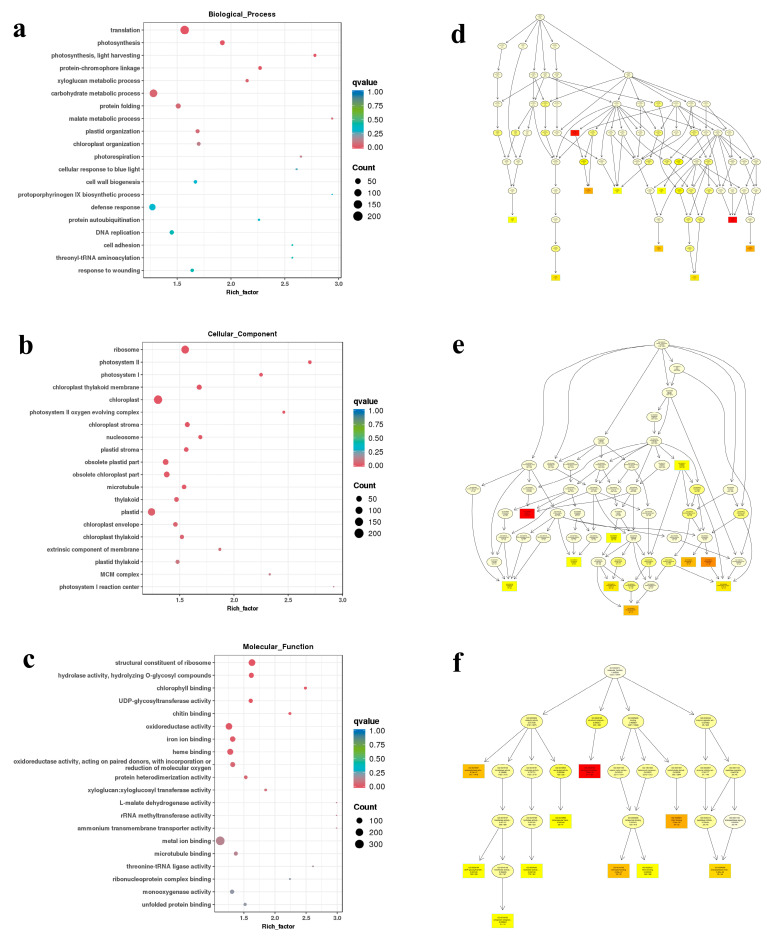
Top 20 pathways in the Gene Ontology (GO) enrichment analysis of DEGs in five comparisons. (**a**) GO enrichment dot plot on Biological_Processes; (**b**) GO enrichment dot plot on Cell_Components; (**c**) GO enrichment dot plot on Molecular_Function; (**d**) GO terms and hierarchical relationship on Biological_Processes; (**e**) GO terms and hierarchical relationship on Cell_Components; (**f**) GO terms and hierarchical relationship on Molecular_Function. Note: Each node represents a GO term, and the box represents the GO with an enrichment level of TOP 5. The depth of the box (or ellipse) color represents the enrichment level, and the darker the color, the higher the significance. The name of the term and the q-value of the enrichment analysis are displayed on each node.

Enrichment analysis was conducted on the DEGs of these five comparative groups, and the enrichment significance of DEGs in GO nodes and KEGG metabolic pathways was obtained. In GO enrichment analysis, the three categories of biological processes with the highest degree of enrichment and difference were photosynthesis light harvesting, malate metabolic processes, and photorespiration. Abundant genes were related to translation, carbohydrate metabolic process, and photosynthesis. Among the top 20 pathways, six were directly related to photoperiod (including photosynthesis, chloroplast organization, and cell response to blue light) (Figure 4a). In the category of cellular components, the three items with the highest enrichment and difference were photosystem II, photosystem I, and photosystem II oxygen-evolving complex, and the three items with the highest number of genes were ribosome, chloroplast, and plastid. Most genes were associated with cell synthesis involved in photosynthesis (Figure 4b); in the molecular functional category, in addition to the structural constituent of ribosome, oxidoreductase activity, chlorophyll binding, and chitin binding all exhibited important enrichment differences (Figure 4c).

The top GO diagrams showed the three main branches of GO function: biological process, cellular component, and molecular function (Figure 4d–f). Photosynthesis and translation were the most enriched terms in the biological process. In the cellular component, ribosome was the most enriched term. In addition, there were multiple pathways pointing toward the chloroplast thylakoid and the photosystem. The molecular function was mainly divided into four pathways: catalytic activity, binding, structural molecules, and enzyme regulation activity. In the pathway of binding, chlorophyll binding was the final result. Taken together, these results indicate that the pathways related to the photoperiod play an important role in the transition from non-bolting to bolting.

**Figure 5:**
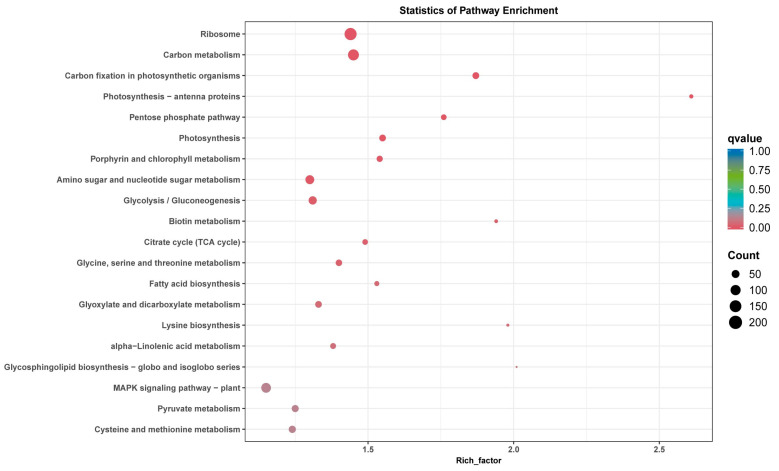
Top 20 pathways in the Kyoto Encyclopedia of Genes and Genomes (KEGG) enrichment analysis of DEGs in five comparisons.

In KEGG enrichment analysis, the photosynthesis antenna proteins pathway had the greatest enrichment and difference, and the pathways with the highest proportion of genes were ribosome, carbon metabolism, amino sugar, and nucleotide sugar metabolism. There were also significant enrichment differences in carbon fixation in photosynthetic organisms, pentose phosphate pathway, photosynthesis, porphyrin, and chlorophyll metabolism in photosynthetic organisms (Figure 5). Several genes in these categories were differentially expressed, which might contribute to bolting time differences between the early- and late-bolting spinach materials.

**Figure 6:**
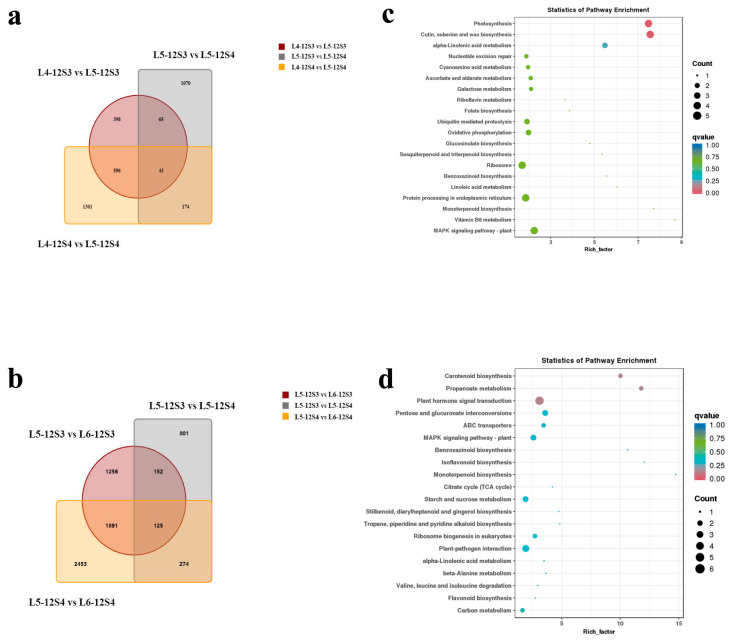
The DEGs at the L4–L5 stage and L5–L6 stage. (**a**) Venn diagram of DEGs in L4-12S3 vs. L5-12S3, L4-12S4 vs. L5-12S4, and L5-12S3 vs. L5-12S4; (**b**) Venn diagram of DEGs in L5-12S3 vs. L6-12S3, L5-12S4 vs. L6-12S4, and L5-12S3 vs. L5-12S4; (**c**) Top 20 pathways in the KEGG enrichment analysis of 174 DEGs in (**a**); (**d**) Top 20 pathways in the KEGG enrichment analysis of 152 DEGs in (**b**).

During the L5 period, bolting-tolerant cultivar 12S3 and susceptible cultivar 12S4 showed obvious differences. Cultivar 12S3 did not bolt during the L5 period, while 12S4 had already bolted. To investigate the reasons for regulating the differences in bolting between these two varieties, we combined and analyzed the DEGs of the L5 stage vs. L4 stage, L5 stage vs. L6 stage, and the DEGs between the two varieties at the L5 stage. We found that 174 DEGs (Figure 6a) and 152 DEGs (Figure 6b) were closely related to bolting at the L4–L5 and L5–L6 stages, respectively.

KEGG enrichment analysis of the 174 DEGs revealed significant enrichment in the pathways of photosynthesis and cutin, suberin, and wax biosynthesis (Figure 6c). The KEGG enrichment analysis of the 152 DEGs showed significant enrichment and differences in the pathways of carotenoid biosynthesis, propanoate metabolism, and plant hormone signal transduction (Figure 6d). These pathways are both important in the process of bolting and may be key pathways that contribute to early and late bolting in different varieties.

### 3.3. Analysis of the Co-Expression Trends of DEGs

**Figure 7:**
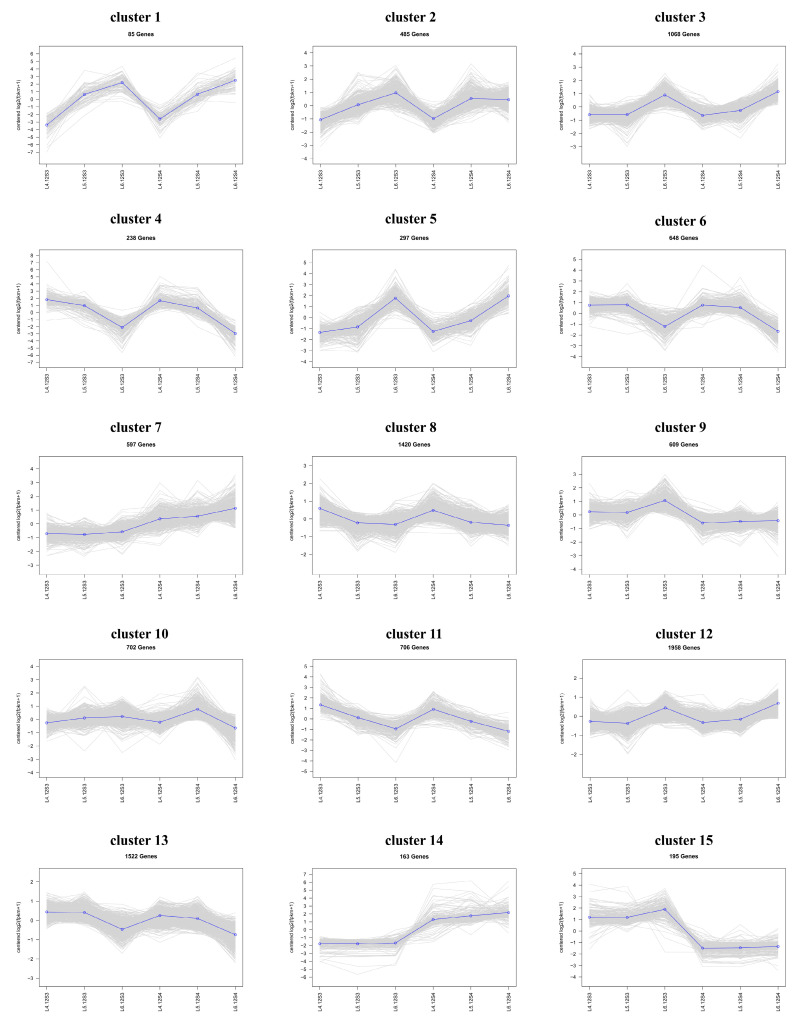
K-means clustering was used to group the expression profiles of the transcriptome into 15 clusters. Gene numbers are shown in each box.

K-means clustering analysis was performed on all DEGs across six groups from three periods with two materials. Based on their dynamic expression, each co-expression gene was classified into 15 clusters (Figure 7). The light grey background color represents the expression of each gene in the cluster, and the blue line in the foreground represents the trend of dynamic expression in all genes in the cluster after fitting the sample data.

Upon observing these 15 clusters, high levels of expression were related to the traits of spinach bolting in clusters 1, 2, and 3. Material 12S3 did not bolt at the L4 or L5 stages but bolted at the L6 stage, and there was a significant upregulation of gene expression from L5 to L6. Material 12S4 did not bolt at the L4 stage but completed bolting at the L5 stage, and there was a significant upregulation of gene expression from L4 to L5. Clusters 1, 2, and 3 showed a clear pattern of change in the materials from unbolting to bolting, and the genes associated with them may play important roles in the bolting process.

The GO enrichment analysis and KEGG enrichment analysis of these three clusters (Supplementary Figures S1–S3) showed a close correlation with pathways such as chloroplast thylakoid membrane, photosystem I, thylakoid membrane, thylakoid lumen, chlorophyll binding, floral whorl morphogenesis, and circadian rhythm–plant, which is similar to the results of the above enrichment analysis of DEGs. This suggests that the trend in bolting-related genes is likely to be similar to that of clusters 1, 2, and 3.

### 3.4. Gene Co-Expression Network Construction

**Figure 8:**
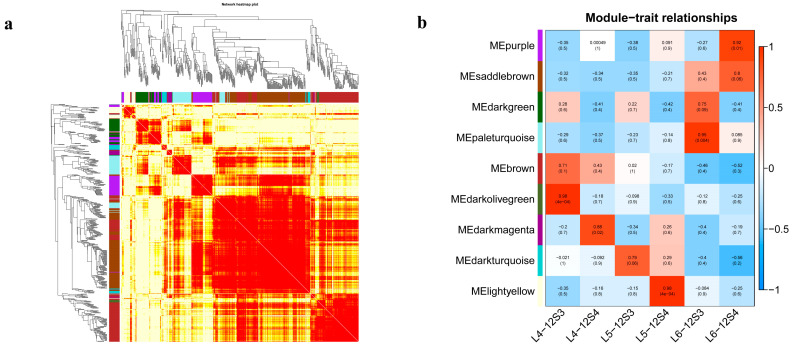
Co-expression modules by weighted gene co-expression network analysis (WGCNA). (**a**) Heatmap of module gene networks; (**b**) Module–trait relationships. Relationships between modules (**left**) and traits (**bottom**). Red and blue represent positive and negative correlations, respectively, with coefficient values and *p*-values.

Based on 5075 genes with fragments per kilobase of exon model per million mapped fragments (FPKM) > 1, nine modules were obtained by WGCNA. The correlation of each module is shown in Figure 8a,b. Upon observing the three stages of 12S4, the modules related to bolting were MElightyellow, MEpurple, and MEsaddlebrown. Upon observing the three stages of 12S3, the most relevant module was MEpaleturquoise. The MEsaddlebrown module was related to both materials.

**Figure 9:**
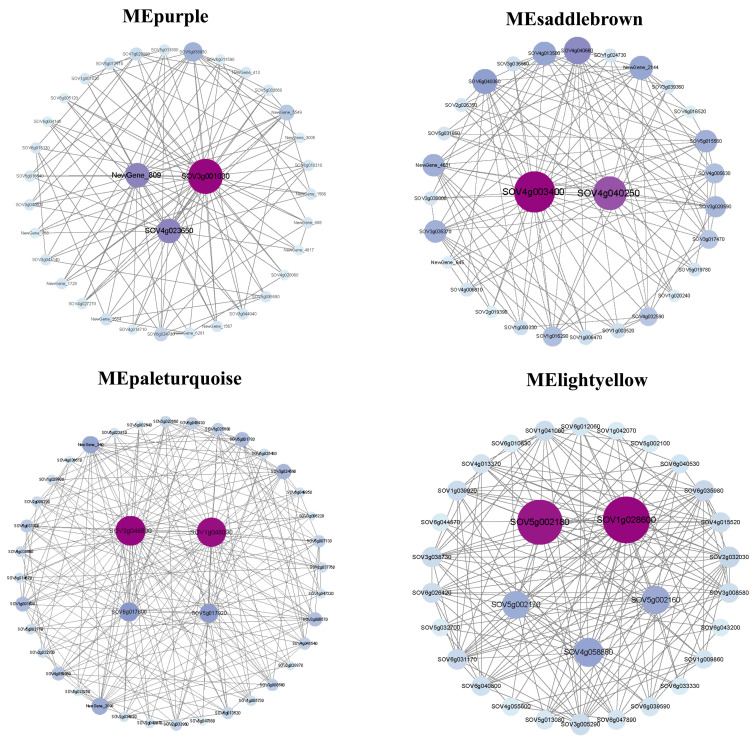
Gene co-expression networks for MEpurple (34 genes, 300 edges), MEsaddlebrown (29 genes, 250 edges), MEpaletuequoise (40 genes, 300 edges), and MElightyellow modules (31 genes, 450 edges).

The analysis of co-expression networks helps to explore the hubs or key genes in each module and their connections to other genes. As illustrated in Figure 9, there were 34 genes and 3 hub genes (*SOV3g001030*, *SOV4g023650*, *NewGene_809*), 29 genes and 2 hub genes (*SOV4g003400*, *SOV4g04025*), 40 genes and 4 hub genes (*SOV3g046800*, *SOV1g046090*, *SOV6g017600*, *SOV5g017920*), and 30 genes and 5 hub genes (*SOV5g002180*, *SOV1g028600*, *SOV5g002170*, *SOV4g058860*, *SOV5g002160*) in the purple module, saddle brown module, pale turquoise module, and light-yellow modules, respectively. These 14 hub genes were highly connected to other genes in these five modules related to bolting, indicating that they play a crucial role in bolting.

On module–trait relationships, MEpaleturquoise was the most highly related module in 12S3 across bolting periods. The following four hub genes were identified: *SOV3g046800*, which belongs to the MAD-box protein family; *SOV1g046090*, which is involved in the cutin, suberin, and wax biosynthesis pathway; *SOV6g017600*, which belongs to the Gibberellin-regulated protein family and is annotated as gibberellin-regulated protein 1-like; and *SOV5g017920*, which remains unannotated. Among these four genes, *SOV6g017600*, the homolog of *GA1*, exhibited the most significant upregulation trend.

MElightyellow, MEpurple, and MEsaddlebrown were highly related modules in 12S4 across bolting and flowering periods. For MEpurple, three hub genes were identified: one (*SOV3g001030*) belongs to the disease resistance protein RGA1, and two (*SOV4g023650*, *NewGene_809*) remain unannotated. For MEsaddlebrown, two hub genes were enriched in circadian rhythm–plant pathways. One gene was annotated as protein *FLOWERING LOCUS T-like* (*SOV4g003400*), and the other was annotated as protein *SPA1-RELATED 4-like isoform X1* (*SOV4g040250*). For MElightyellow, three genes belonged to the Potato inhibitor I family (*SOV5g002180*, *SOV5g002170*, and *SOV5g002160*) and were enriched in the cutin, suberin, and wax biosynthesis pathways. Two additional genes, one belonging to the Early Flowering 4 domain (*SOV1g028600*), encoding protein *EARLY FLOWERING 4-like*, and one in the GRAS domain family (*SOV4g058860*) were involved in the synthesis pathway of DELLA protein *RGL1*.

Utilizing the abbreviation ‘Sp’ for spinach and pairing it with common gene symbols to represent these genes, along with providing brief annotations, the information for these hub genes is presented in Table 1.

**Figure 10:**
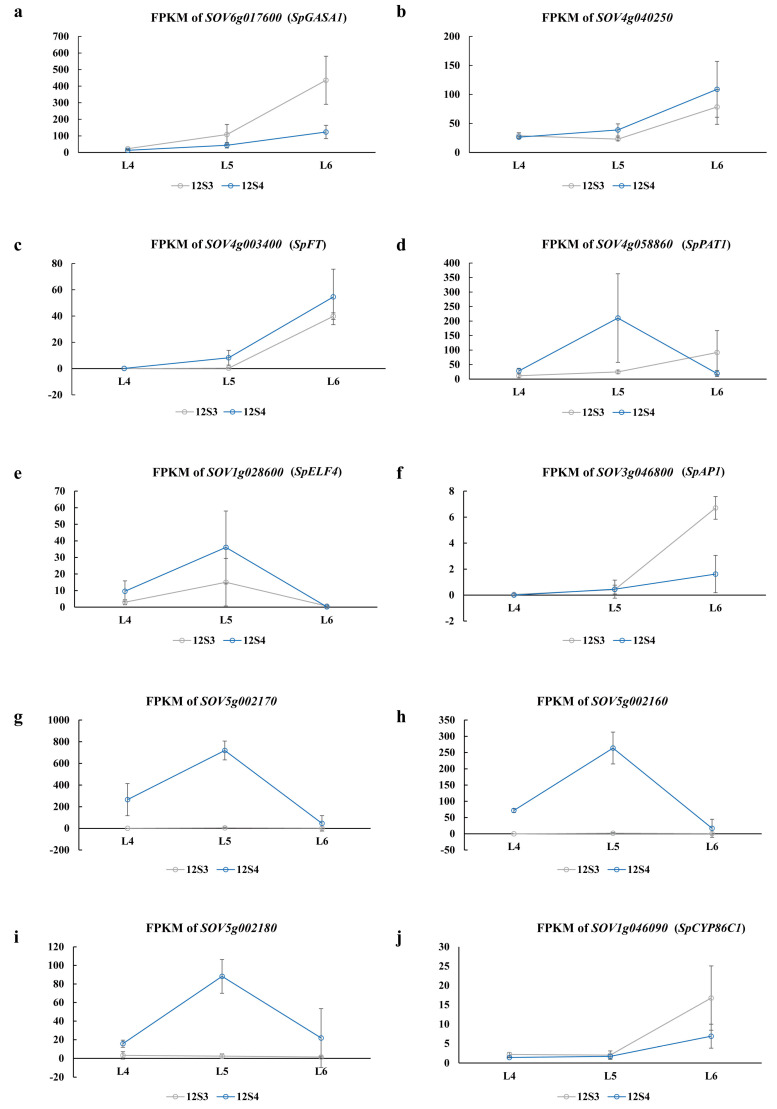
Changes in the expression of related genes at different bolting stages, using fragments per kilobase of exon model per million mapped fragments (FPKM) value as the standard for expressing changes in quantity. (**a**) FPKM of *SOV6g017600*; (**b**) FPKM of *SOV4g040250*; (**c**) FPKM of *SOV4g003400*; (**d**) FPKM of *SOV4g058860*; (**e**) FPKM of *SOV1g028600*; (**f**) FPKM of *SOV3g046800*; (**g**) FPKM of *SOV5g002170*; (**h**) FPKM of *SOV5g002160*; (**i**) FPKM of *SOV5g002180*; (**j**) FPKM of *SOV1g046090*.

Combined with the annotation information, the expression of the above hub genes in different varieties at different times is shown in Figure 10. *SOV6g017600* (*SpGASA1Gibberellin-regulated protein 1*), *SOV4g003400* (*SpFT*), *SOV4g040250* (*SPA1-RELATED 3*), *SOV3g046800* (*SpAP1*), and *SOV1g046090* (*SpCYP86C1*) showed a clear trend of upregulation (Figure 10a–c,f,m)*. SOV5g002160*, *SOV5g002170*, and *SOV5g002180*, which are involved in cutin, suberin, and wax biosynthesis, had the same expression trend: no significant change in expression in 12S3, upregulation in 12S4 in the L5 period, and downregulation in the L6 period (Figure 10h–j). The expression of the *Early Flowering* gene *SOV1g028600* (*SpELF4*) was upregulated at the L5 period and downregulated at the L6 period (Figure 10e). *SOV4g058860* (*SpPAT1*) is a homologue of the DELLA protein *RGL1*, and interestingly the period of its upregulation corresponded to the transition from no bolting to bolting (Figure 10d).

### 3.5. qRT-PCR Validation of Major Hub Genes

**Figure 11:**
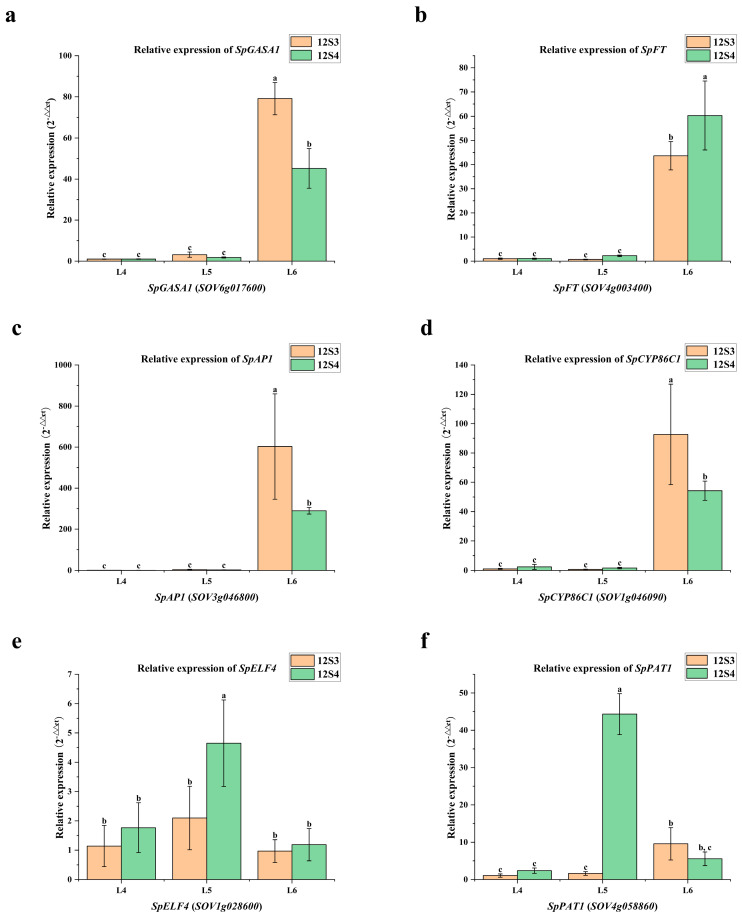
qRT-PCR was performed using six major hub genes. Values with the same letter were not significantly different at *p* < 0.05. (**a**) Relative expression of *SpGASA1*; (**b**) relative expression of *SpFT*; (**c**) relative expression of *SpAP1*; (**d**) relative expression of *SpCYP86C1*; (**e**) relative expression of *SpELF4*; (**f**) relative expression of *SpPAT1*.

The transcription levels of six hub genes, identified by common gene symbols, were assessed using qRT-PCR (Figure 11). The RNA-seq and qRT-PCR results for these six genes were consistent, indicating the reliability of the high-throughput transcriptome sequencing. It can be seen from the intuitive result map that *SpELF4* and *SpPAT1* exhibit significant expression at the L5 stage in the 12S4 material, while their expression is not significant during the L5 stage in the 12S3 material. This discrepancy indicates a potential correlation between these two genes and the divergence in bolting timing observed in these two materials.

## 4. Discussion

Spinach is a vegetable that uses green leaves as its main product organ. Bolting time has a significant impact on its production. Different spinach varieties exhibit significant differences in bolting behavior, with bolting-tolerant varieties often having longer vegetative growth periods, indicating greater potential for marketability. Based on our previous investigations of phenotypic data, 12S3 was defined as a bolting-tolerant material (an average bolting time of 62 days), and 12S4 was defined as a bolting-susceptible material (an average bolting time of 46.5 days). We sampled both materials at three stages: L4, L5, and L6. At the L4 stage, neither material was bolted. At the L5 stage, 12S4 was bolted, and 12S3 was not bolted. At the L6 stage, both materials were bolted. RNA-seq was performed on the different materials at different stages for functional enrichment analysis, gene co-expression trend analysis, and gene co-expression network analysis.

In this study, a significant number of differentially expressed genes (DEGs) were involved in the photoperiod pathway, affecting processes such as photosynthesis and the formation of the photosystem, chloroplasts, and chlorophyll binding. A smaller number of DEGs were associated with plant hormone signal transduction, floral whorl morphogenesis, and circadian rhythm in plants.

A GO enrichment analysis revealed that translation, carboxylate metabolic processes, and photosynthesis account for the most DEGs in biological processes. The translation process involving ribosomes synthesizes a large number of proteins for biological processes. Carbohydrates regulate nutritional and reproductive growth, not only as an energy reserve for inflorescence growth but also as a signaling molecule [31]. Spinach is typically a photosensitive plant, and LD can promote bolting and flowering. The timing of bolting can be regulated during spinach cultivation by controlling the length of daylight and temperature using artificial light [32]. In the category of cellular components and molecular function, photosystem, ribosome, and chlorophyll binding showed conspicuous differences and enrichment. Multiple pathways pointed toward chloroplast thylakoid, photosystem, and chlorophyll binding. Several unigenes in these categories were differentially expressed, which might explain the difference in bolting times between 12S3 and 12S4.

In KEGG enrichment analysis, many genes involved in the ribosome and carbon metabolism pathways, as well as pathways related to photosynthesis, showed significant enrichment. This was similar to the results of GO enrichment. When comparing the L5 period, which showed differences in bolting alone, with the other two periods, the KEGG results showed significant enrichment in the pathways of photosynthesis, cutin, suberin, and wax biosynthesis, carotenoid biosynthesis, propanoate metabolism, and plant hormone signal transduction. Several of these pathways may be the key pathways responsible for the differences in early and late bolting.

We conducted a co-expression trend analysis and found that the trends of the three clusters were similar to the changes in bolting traits, and the gene enrichment of these three clusters was consistent with the previous enrichment analysis. It is speculated that the trends of these three clusters are the expression trends of bolting-related genes. The GO enrichment analysis and KEGG enrichment analysis of these three clusters showed a close correlation with pathways such as chloroplast thylakoid membrane, photosystem I, thylakoid membrane, thylakoid lumen, chlorophyll binding, floral whorl morphogenesis, and circadian rhythm–plant, which was similar to the results of the previous enrichment analysis of DEGs.

We then combined WGCNA analysis and gene annotation to obtain some interesting hub genes. Genes *SOV4g003400* (*SpFT*) and *SOV4g040250* are involved in the circadian rhythm–plant pathway. The former is a homolog of the flowering gene *FT* in Arabidopsis [11], and the latter is involved in the generation of phytochrome A (PhyA), an *SPA* protein that influences plant growth and flowering by regulating photosynthesis and mitochondrial function [33,34]. Their expression gradually increases with longer light exposure. In bolting-susceptible variety 12S4, the expression of these genes was higher during the same period compared with bolting-tolerant variety 12S3 (Figure 10b,c). *FT* is an important node gene in the photosignaling and vernalization pathways of flowering regulation in Arabidopsis, and its function is conserved in different flowering plants. However, induction of the expression of *Hd3a*, a homologous gene of *FT*, in rice promotes early tasseling in rice under short-day conditions [35]. PhyA can promote the flowering process of plants under LD conditions [36]. These two genes may play a positive regulatory role in the bolting process.

*SOV3g046800* (*SpAP1*) and *SOV1g046090* (*SpCYP86C1*) are two other genes that show an upregulation trend (Figure 10f,m). Overall, the expression of these two genes was higher in bolting-tolerant variety 12S3 than in bolting-susceptible variety 12S4, indicating significant varietal differences.

*SOV1g028600* (*SpELF4*) is an early flowering gene involved in the positive regulation of circadian rhythm in biological processes (Figure 10e). At the L5 stage, the expression of *SOV1g028600* (*SpELF4*) in bolting-susceptible material 12S4 was significantly higher than in bolting-tolerant material 12S3, suggesting its involvement in the early bolting process.

*SOV6g017600* (*SpGASA1*) and *SOV4g058860* (*SpPAT1*) are involved in plant hormone signal transduction. The former is a gibberellin-regulated protein 1-like gene, and it showed an upregulation trend during the process of bolting, with higher expression in 12S3 (Figure 10a,d), suggesting that bolting-tolerant varieties may require more gibberellins to initiate flowering. The other gene belongs to the *GRAS* domain family and is involved in the synthesis of the DELLA protein *RGL1*. *RGL1* is a gibberellin (GA) inhibitor that plays a role in the negative regulation of the GA signal [37,38]. At low GA concentrations, DELLA proteins inhibit flowering by suppressing GA; when GA levels increase, DELLA is degraded, allowing the plant to initiate bolting and flowering [38]. In 12S4, bolting occurred at the L5 stage, with the transition to flowering completed at L6. *SOV4g058860* (*SpPAT1*) was highly expressed at L5 but significantly downregulated at L6, indicating that this gene may cease its response to gibberellins after bolting is completed, which aligns with the high expression of *SOV6g017600* (*SpGASA1*) at L6. In 12S3, bolting just started at L6, with some time remaining before flowering. Both *SOV4g058860* (*SpPAT1*) and *SOV6g017600* (*SpGASA1*) were highly expressed at L6, suggesting that they may play a role in suppressing premature flowering, while bolting is in progress.

The cutin, suberin, and wax biosynthesis pathways, which include *SOV5g002160*, *SOV5g002170*, and *SOV5g002180*, are frequently mentioned and are speculated to play a role in the transition from nutritive to reproductive growth in spinach. These three genes were highly expressed during the bolting period of 12S4 and very lowly expressed during the same period of 12S3 (Figure 10h–j), which may be related to the early bolting of 12S4.

## 5. Conclusions

Bolting is an important trait that affects the quality and economic efficiency of spinach, and the study of bolting mechanism is of great significance for spinach production. To date, studies on the mechanisms related to bolting of spinach have focused only on the comparison between two stages (the bolted and unbolted), and related research approaches have been relatively narrow. In this study, RNA-seq was performed using two typical spinach materials of bolting-tolerant and bolting-susceptible, and the enrichment of differentially expressed genes, co-expression trends, and co-expression networks were analyzed to reveal the key pathways and hub genes leading to spinach bolting. The results showed that the photoperiod and gibberellin pathways were the key pathways of spinach bolting. Differences in early and late bolting in different varieties may be related to the signal transduction of the cutin, suberin, and wax biosynthesis pathway and the gibberellin-related signaling pathway. *SpFT* (*SOV4g003400*) and *SOV4g040250* are the key genes in photoperiodic pathway, and *SpGASA1* (*SOV6g017600*) is the key gene in gibberellin pathway, all of which have a significant influence on spinach. *SpPAT1*(*SOV1g028600*) and *SpELF4* (*SOV4g058860*) may be the key genes contributing to bolting-tolerance and bolting-susceptibility of different varieties. In conclusion, the results of this study provide the most complete transcriptomic data available on spinach bolting and identify candidate hub genes in the bolting process, which can be used for future breeding studies.

## Figures and Tables

**Table 1:** The gene IDs and their corresponding gene symbols for these hub genes.

Gene ID	Gene Symbol	Annotation
*SOV6g017600*	*SpGASA1*	*Gibberellin-regulated protein 1 (GAST1 protein homolog 1)*
*SOV4g003400*	*SpFT*	*Flowering Locus T*
*SOV4g040250*	NA	*Suppressor of PhyA-105 RELATED 3 (SPA1-RELATED 3)*
*SOV3g046800*	*SpAP1*	*Agamous-like MADS-box protein AP1*
*SOV1g046090*	*SpCYP86C1*	*Cytochrome P450*, *family 86*, *subfamily C*, *polypeptide 1*
*SOV5g002160*	NA	*Serine Protease Inhibitor*
*SOV5g002170*	NA	*Serine Protease Inhibitor*
*SOV5g002180*	NA	*Serine Protease Inhibitor*
*SOV1g028600*	*SpELF4*	*Early Flowering protein 4*
*SOV4g058860*	*SpPAT1*	*Phytochrome A Signal Transduction 1 (PAT1)*

“NA” stands for “Not Applicable”.

## Data Availability

Data are contained within the article and Supplementary Materials.

## Supplementary Materials

The following supporting information can be downloaded at https://www.mdpi.com/article/10.3390/genes15010036/s1. Table S1: Summary of Illumina RNA-seq data; Table S2: Primer sequences for qRT-PCR; Figure S1: Top 20 pathways in the GO and KEGG enrichment analysis of DEGs in Cluster 1; Figure S2: Top 20 pathways in the GO and KEGG enrichment analysis of DEGs in Cluster 2; Figure S3: Top 20 pathways in the GO and KEGG enrichment analysis of DEGs in Cluster 3.
