# Supplementary figures and images for: Comparative Transcriptome Analysis of Gene Expression and Regulatory Characteristics Associated with Different Bolting Periods in Spinacia oleracea
Source: Genes (Basel). 2023 Dec 26;15(1):36. doi: 10.3390/genes15010036 (PMC10815260; doi:10.3390/genes15010036)

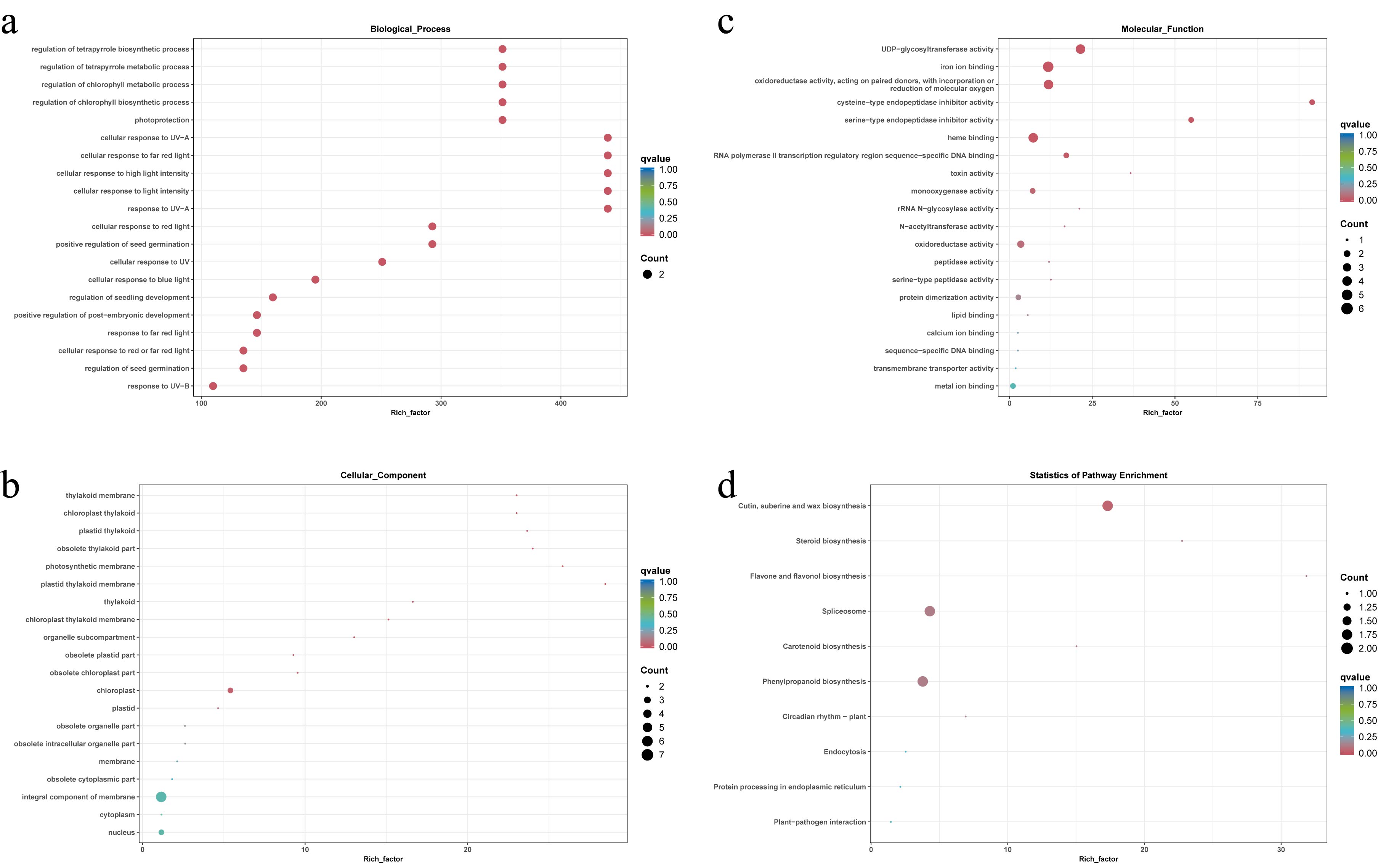

Supplement: Supplementary file 1 [file genes-15-00036-s001.zip › Figure S1.jpg]

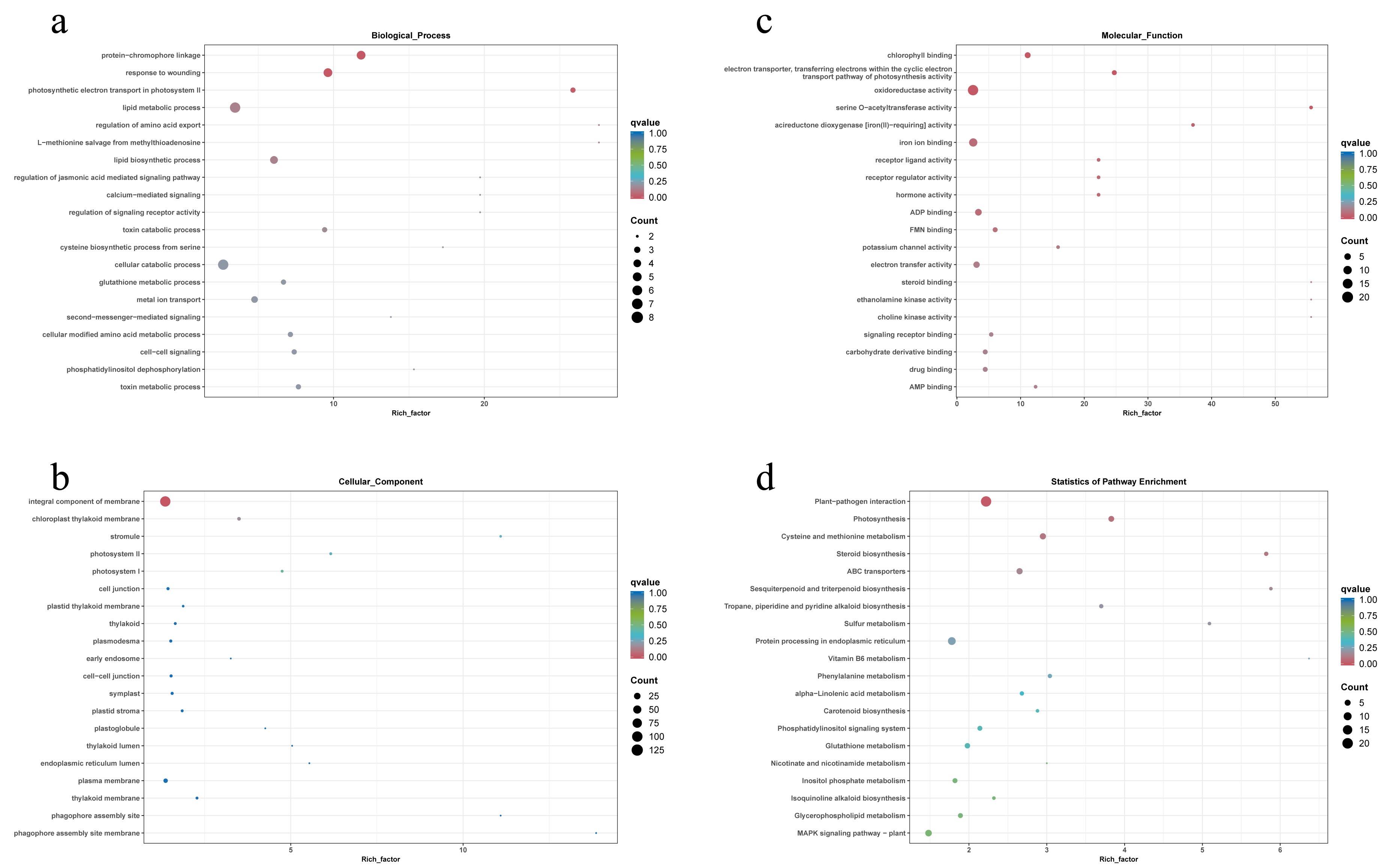

Supplement: Supplementary file 1 [file genes-15-00036-s001.zip › Figure S2.jpg]

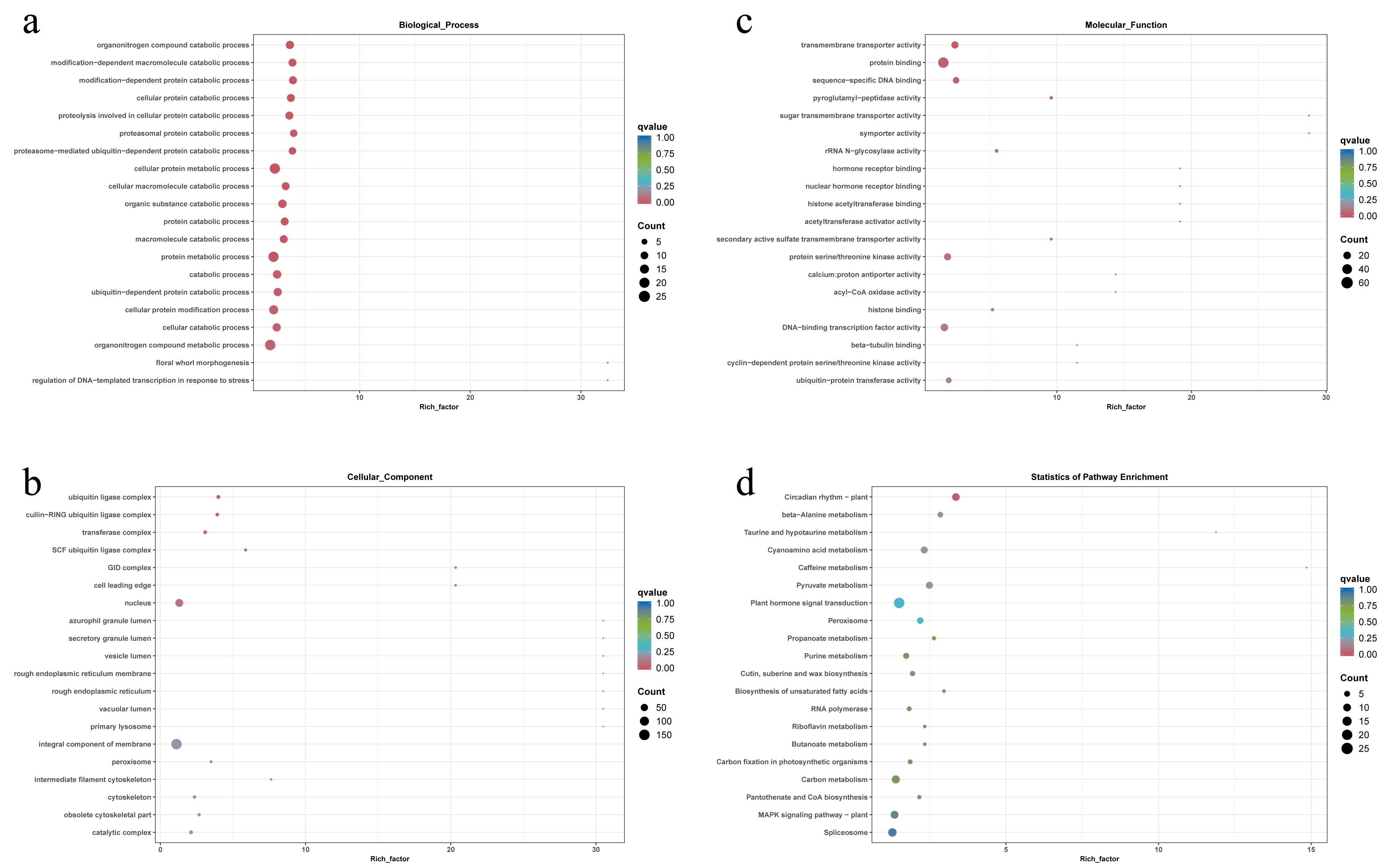

Supplement: Supplementary file 1 [file genes-15-00036-s001.zip › Figure S3.jpg]
